# Digitally enabled supply chain as a strategic asset for the COVID-19 response in Alberta

**DOI:** 10.1177/08404704211057525

**Published:** 2022-03

**Authors:** Anne Snowdon, Alexandra Wright

**Affiliations:** 1 8637 University of Windsor, Windsor, Ontario, Canada.; 2 University of Toronto, Toronto, Ontario, Canada.

## Abstract

This provincial case study, one of seven conducted as part of a national research program on healthcare supply chain management during COVID-19, focuses on Alberta. With a history of emergency preparedness, Alberta’s unique context, one that includes having an already established, centralized, and digital healthcare supply chain strategy, sets this case apart from the others in terms of pandemic responses. A key challenge navigated by Alberta was the inadequacies of traditional sourcing and procurement approaches to meet surges in product demand, which was overcome by the implementation of unique procurement strategies. Opportunities for Alberta included the integration of supply chain teams into senior leadership structures, which enabled access to data to inform public health decision-making. This case demonstrated how Alberta’s healthcare supply chain assets—its supply chain infrastructure, data, and leadership expertise, especially—contributed to resilient supply chain capacity across the province.

## Introduction and review of the literature

The COVID-19 pandemic has shone a bright light on the critical importance of healthcare supply chain as a strategic asset that enables safe work environments for the health workforce and quality care delivery for Canadians. Supply chain and logistics infrastructure is a strategic asset in health systems that makes it possible to respond to unexpected events, such as surge in demand for care during a pandemic, to ensure that healthcare workers have the products and equipment necessary to deliver care during surge events to achieve the best possible outcomes for patients. Supply chain in health systems includes the sourcing and distribution of products that ensures healthcare teams have access to the right products at the right time, in order to deliver safe and effective patient care.^1^ In health systems, supply chain teams source a complex and diverse array of products and equipment, from ventilators and intravenous pumps, to medications, vaccines, and Personal Protective Equipment (PPE).

Supply chain disruptions can occur due to natural disasters and public health crises, resulting in severe consequences that put health workers and patients at significant risk. For example, during Hurricane Maria in 2017, electrical grids were wiped out in Puerto Rico, which impacted the production of IV bags manufactured by Baxter.^2^ The resulting shortage in supply of IV bags skyrocketed globally, causing an increase in cost by 600%.^2^ Similarly, a flood in 2012 impacted Sanofi Pasteur, the supplier of the cancer drug ImmuCyst. The result was significant delays in cancer treatment to patients as manufacturers could not increase production of the drug rapidly enough to meet the demand.^3^ Public health crises, such as the SARS epidemic, also put pressure on health system supply chains, due to increase in demand for critical products to keep health workers and patients safe. These crises put the physical health of essential workers at risk—three of the 44 Canadians who died from SARS were healthcare workers,^4^ and emerging evidence identifies significant impact on the mental health of the workforce when real or perceived supply shortages occur during these public health events. The inadequate supply of PPE and the uncertainty these supply shortages create among Canada’s healthcare workforce directly impacted their mental health during the COVID-19 pandemic.^5^

Healthcare supply chain management and infrastructure lag significantly behind the well-developed supply chains in other business sectors.^6-8^ Supply chain and logistics have been well researched and developed in the private sector; however, significant gaps in research still exist in the healthcare sector.^9-12^ One reason for the paucity of research in this sector is the fact that there are features that are unique to healthcare supply chain that do not apply across sectors. Healthcare supply chains involve many more stakeholder groups compared with other business sectors, including patients, clinicians, suppliers, healthcare organizations, group purchasing organizations, distributors, and insurers, making them highly complex systems.^13,14^ Both the range and complexity of products in healthcare supply chain are also unique when compared with other sectors. Unique features of the healthcare supply chain make it particularly complex,^13^ limiting the applicability of supply chain research from other sectors to healthcare.^10^ Of most significance perhaps is when the healthcare supply chain breaks down, the result is a direct impact on human life.^15-17^ The COVID-19 pandemic has highlighted the urgent need for healthcare supply chain specific research, to further understand the nuances evident in this sector.

This paper reports on case study research of the province of Alberta’s response to the COVID-19 pandemic, revealing empirical evidence of supply chain processes and infrastructure within and across the provincial health system, during the first two waves of the pandemic. This case study of Alberta is one of seven conducted to examine health supply chain capacity and infrastructure across Canada, the first national study of health supply chain, funded by CIHR (Ref. VR5 #172669). The following case study examines supply chain processes and infrastructure and the impact of supply chain capacity on healthcare delivery in Alberta, and how it contributed to COVID-19 outcomes. The following research questions were examined:

• What are the supply chain processes and infrastructure required to optimize effective and timely health services delivery for the current and future phases of the COVID-19 pandemic?
• What procurement models, approaches, and policy frameworks offer secure sourcing of products to meet the surge in demand for care by COVID-19 patients?
• What is the digital maturity of supply chain infrastructure (and processes) in Alberta, that, if strengthened, could optimize management of COVID-19?
• What are the data infrastructure and analytics strategies needed to strengthen the effectiveness of health system supply chain processes to support COVID-19 management?
• What is the influence of federal government initiatives, from the perspective of provincial stakeholders, on provincial health system capacity to manage COVID-19?

## Methods

A case study research approach was employed to examine the capacity of healthcare supply chain in Alberta, given the dearth of research on healthcare supply chain in Canada to date. The University of Windsor’s Research Ethics Board provided approval for this project. This case was one of seven, as part of a national CIHR Rapid Research program (CIHR Ref. #VR5 172669) entitled “*Development of an Implementation Framework to Advance Provincial and National Health System Supply Chain Management of COVID-19*.” A case study approach was used to understand Alberta’s healthcare supply chain response in COVID-19. Case studies offer a way to explore and investigate real-life phenomenon through analyzing the context of events and the relationships between them.^18^ The primary data source for this study came from 13 semi-structured interviews of health system stakeholders as well as critical review of public documents and reports. Data collection was guided by a semi-structured interview guide examining supply chain capacity and processes and their relationship to decisions and management of the pandemic, completed following the second wave of the COVID-19 pandemic. Key participants who represented varied perspectives and expertise including leaders in supply chain, procurement, clinician leaders (e.g., physicians, nurses, pharmacists, and primary care), health executives, union leaders, industry leaders, and government were selected using purposive sampling initially and then snowball sampling as leaders identified informants to be approached to participate in the study. Semi-structured interviews documented the experiences, perspectives, and views of how supply chain infrastructure and processes were operationalized during the early waves of the pandemic, the impact of leadership decisions on supply chain management, how supply chain capacity influenced leadership decisions and COVID-19 health system outcomes, and how and which challenges, solutions, and gaps in supply chain infrastructure contributed to COVID-19 outcomes. Interviews were conducted until such time as theoretical saturation was achieved. Coding of interviews proceeded as data was collected, whereby researchers were able to identify emerging themes and concepts to enable reflexivity as interview data were collected and analyzed. Interviews were audio-recorded using Microsoft Teams and transcribed verbatim by an independent transcriptionist. The analysis included detailed and multiple review of transcripts to identify initial concepts, followed by coding, and categorizing text excerpts to identify conceptual patterns across transcripts that were developed into themes, using N-Vivo software to assist with organization of data. The themes were then defined, and are described in the following sections.

### Provincial context

Located in Western Canada, the province of Alberta has a population of over 4 million people, with a majority clustered around the major urban centres of Calgary and Edmonton. Alberta Health Services (AHS) is Canada’s first and largest province-wide, integrated healthcare system, providing healthcare services to over 4.4 million people.^19^ In 2008, Alberta amalgamated all of its health regions into one provincial organization, AHS. Alberta Health Services is responsible for all hospital services and health services in a number of health organizations including palliative care, mental health and continuing care, and some long-term care organizations.^20^ The consolidation of all regional health authorities into one entity created an “integrated healthcare system” that allowed AHS to share information and provide standardized care across many healthcare organizations.^20,21^ Currently, AHS has 106 acute care hospitals, care delivery programs at 850 facilities, 27,774 continuing care beds, 256 community palliative and hospice beds, and 2,785 addiction and mental health beds.^19^ The goal of a more centralized health system was the reduction in duplication of services and functions across the many health regions and agencies (e.g., supply chain and procurement processes, policy and accountability processes, HR, and finance). A consolidated management structure has enabled Alberta to advance a province-wide data infrastructure both within and across health organizations in all regions of the province. Alberta’s province-wide governance model and centralized data infrastructure measure health system performance across the province.

When AHS was created, and provincial healthcare was centralized, Alberta made a significant investment in its enterprise resource planning infrastructure, which enabled digital supply chain infrastructure and visibility of product utilization for all hospitals across the province. This data infrastructure enables tracking and traceability of product utilization from manufacturers to patient care units in each hospital, including the adoption of global standards to support accuracy of product identification and product attributes.^23^

Alberta Health Services has established the Contracting, Procurement, and Supply Management (CPSM) team to manage all supply chain processes across the province. The CPSM team is responsible for the contracting, purchasing, inventory management, warehousing, and distribution of supplies, products, and equipment.^24^ The mandate of this team is to manage supply chain services for all AHS organizations, including sourcing, procuring products, tracking utilization, integrating clinicians into supply chain teams, and managing supply chain data.^24^ Prior to the COVID-19 pandemic, the CPSM supply chain team, and AHS more broadly, had reported significant gains in cost savings for supplies by streamlining supply processes, tracking utilization and product inventory, and reducing costs due to product waste. All sourcing, contracts, and procurement are managed by the CPSM team, ensuring every hospital and care team have the products needed to deliver care.

### COVID-19 in Alberta

**Figure 1.**
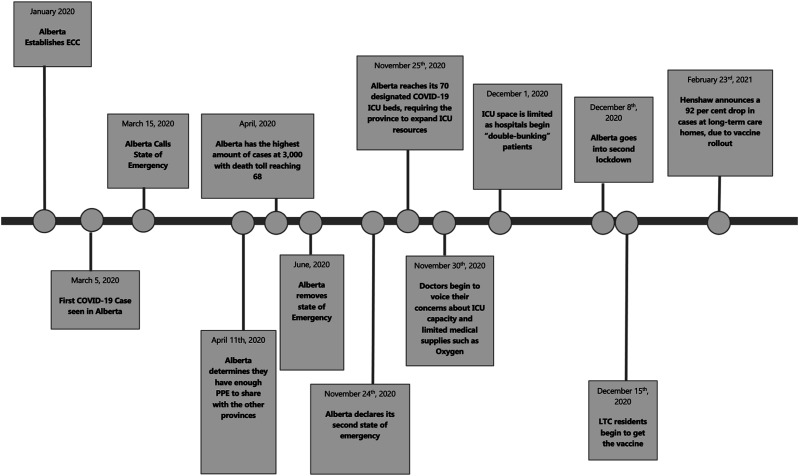
Sequence of events in Alberta.

The first Canadian case of COVID-19 was reported January 26, 2020 in Toronto, Ontario, with cases rapidly spreading across the country in the following months.^25^ On January 30, 2020, the province of Alberta Emergency Coordination Centre (ECC) was established to manage the pandemic response across the province. On March 5, 2020, Alberta reported its first case of COVID-19 and public health measures were quickly established to contain the spread of the virus. Alberta responded quickly to the first wave of the pandemic, announcing a state of emergency on March 15, 2020 and implemented the necessary public health restrictions.^25^ During the first wave of COVID-19, cases in Alberta peaked at 3,138 on October 19, 2020.^26^ In May 2020, the province saw a decline in new infections, and restrictive public health measures were eased. In November 2020, things again began to change as case counts began to rise. In the second wave, cases hit a peak of 20,500 active cases.^27^ The rapid rise in case numbers of COVID-19 overwhelmed Alberta’s contact tracing system, which had limited human resources capacity to keep up with the demands for lab testing services.^28^ As the second wave unfolded, the government delayed implementation of additional restrictions or public health initiatives, particularly lockdowns, despite rising case counts.^29^ Clinicians began to voice their concerns about the capacity of the health system to meet demands for care, including concerns for the limited health workforce resources and critical supplies, such as oxygen, to adequately support patient care.^30^ Field hospitals were created to increase hospital capacity, and several intensive care units were required to “double-bunk,” putting two patients in each room to manage the increasing numbers of critically ill patients infected with COVID-19.^31^ As the province reached 1,500 new cases a day, Alberta reached out to the Federal Government and Red Cross for support of the field hospitals.^32^ Alberta entered its second lockdown on December 8, 2020, as vaccination programs began in long-term care organizations. December saw the highest number of cases of COVID-19 among long-term care residents, with 776 cases reported, and two out of three COVID-19 deaths were residents of long-term care.^33^ To reduce the impact on long-term care, Alberta began to prioritize the vaccination of long-term care residents. Just two months later, on February 23, 2021, the Chief Medical Officer of Health announced that there had been a 92% drop in cases at long-term care homes, attributed to the vaccine roll-out.^34^ The sequence of key events is summarized in Figure 1. The key themes emerging from analyses profiled the critical role of leadership and governance structures that supported leader decision-making approaches to manage the pandemic.

### Leadership, governance structure, and decision-making

The Minister of Health and Premier of Alberta were the primary decision-makers for Alberta’s COVID-19 response, informed and advised by public health and AHS leadership, described by one key informant:

*“Policy decisions are made by government cabinet and we deliver… we have an ability to influence, but government is owning everything.”* (Health System Leader)

Findings revealed a decision-making strategy that was centralized in the Alberta government whereby AHS leadership provided information to inform decisions, described by one key informant:

*“What government did early on was they set up something called the emergency management cabinet committee. This emergency management cabinet committee, we call it EMCC, at one point was meeting three times a week. AHS would be there probably about 80% of the time. (Supply chain leaders) would be present. It was chaired by the Premier, and that was where a lot of the decisions were made about shutting down schools, social distancing, visitation guidelines, so a lot of the operational pieces. They wanted reporting from AHS, and you know, PPE was part of that.”* (Supply Chain Leader)

Alberta Health Services provided supply chain data and information to the cabinet committee in order to provide supply chain expertise to government. A key informant described how supply chain data informed government decisions:

*“…the emergency management committee of the cabinet was presented with the options in our model which basically said, you know, in a probable scenario this is what you’re going to need. In each scenario, he was provided with information on what (supplies) were available today, and the projected burn rate, and how many days was that going to last.”* (Health System Leader)

Alberta had previous experience with emergencies, such as wildfires (e.g., Fort McMurray) and severe floods (Calgary), which meant that emergency management plans, communication, and governance structures were well established in Alberta, well before the COVID-19 pandemic. Key informants identified that experience with the history of crises in Alberta had established the emergency management structures necessary to respond to the COVID-19 crisis. A key informant described this as “organizational resilience,” in the following:

*“You have to think about the fact that it’s not just about personal resilience. It’s everybody has got to have their personal resiliency built up, but it’s also about organizational resiliency. So, if you as an organization are resilient with your, you know, for example being able to have a good emergency disaster management system in place, making sure you've got business continuity, making sure you've got the workforce. That organizational resiliency is I think what’s been key, I think, to keeping people functioning.”* (Health System Leader)

Although Alberta was familiar with crisis management, the pandemic required leadership to adapt to the rapidly changing events. A key informant describes the leadership’s ability to learn and adapt to the rapidly changing pandemic scenarios:

*“We were at some point changing policy every week or every two weeks. I think just the really rapidly changing environment. You know we also learned pretty quickly that the best way to deal with issues, with PPE, we did a PPE task force. That management organization model, we had to change our emergency coordinating center model to something that was more adaptable for the environment that we’re in… We put these task forces together, bring all the experts across the province and then we were much more nimble at approaching things.”* (Health System Leader)

A task force strategy, such as the PPE task force, was created to mobilize expertise across the province to further inform leadership decisions to manage the pandemic. The Alberta Strategic Clinical Networks (SCNs) were engaged to inform decisions as the SCN’s were structured to integrate clinical expertise, research capacity, patient perspectives, and policymakers that were accessed to generate evidence inform task force decisions, described by a key informant:

*“In Alberta you know, we always took a provincial lens and even before pandemic hit, several of our (Strategic Clinical) networks were involved in pulling together provincial groups to start to organize ourselves provincially around what we thought we would need to do during COVID 19… There was a PPE task force that was set up, so how do we think about making sure we have PPE, and not only do we have it, can we build a production model so that we can anticipate our needs over time.”* (Health System Leader)

Pandemic response and management were led by government cabinet, informed by AHS data, information, and leader expert advice. The role of AHS leadership was to operationalize government decisions to manage the pandemic. The province-wide leadership structure reached across clinician leaders, academic leaders, and patients by mobilizing existing SCNs across the province. The existing leadership infrastructure of AHS supported a highly integrated and centralized provincial pandemic response, led by government decision makers.

### Supply chain visibility and provincial capacity

Alberta Health Services had an established supply chain digital infrastructure at the onset of the pandemic, which enabled tracking and traceability of critical supplies and equipment from manufacturer or supplier to hospital care settings. Implementation of a province-wide Electronic Medical Record (EMR) was underway at the time the pandemic unfolded in Alberta. However, until the EMR installation was completed, traceability of products was visible only to clinical care units. Point of care capture of product utilization was not yet possible, which precluded visibility of product utilization at the point of care. However, the existing digital infrastructure enabled Alberta leaders to track supply utilization rates for every hospital care setting in the province:

*“We knew that pre-COVID our burn rate was about 33,000 a day. At kind of the peak of COVID when we had the highest number of cases, we were running through 760,000 a day.”* (Supply Chain Leader)

The Alberta team had members with close relationships in China, which offered early insights into the unfolding pandemic well before COVID-19 cases were evident in North America, described in the following:

*“I think, it was probably December, that we had started having some discussion on what the impact [of COVID-19] would be. It was mostly because we had people who work with us who are Chinese and are still very well connected back home…. What we did do at that point in time was review what our stockpile was, of course never anticipating that the demand was going to be so significant, and basically made a request to purchase about $2.3 million dollars’ worth of inventory of some of the basic needs, like at that time we thought we should order N95s.”* (Health System Leader)

The global shortage of critical supplies quickly escalated. Alberta had the advantage of accurate utilization data, described as the “burn rate,” which informed preparations on managing product shortages, particularly PPE. As the pandemic progressed, the CPSM team began to experience increased difficulty procuring supplies as every global health system competed for supplies in critical shortage. A key informant describes the limitations of the supply chain strategies, which prioritized lowest cost, rather than supply diversity and capacity:

*“I think failing of the supply chain in healthcare systems is because of the real focus on reduced costs and therefore you know, streamlining the supply chain channels and you know, those types of things, we’re also tied into these contracts with GPOs.”* (Health System Leader)

Traditional procurement strategies, such as bulk purchasing through group purchasing organizations (GPOs), were unable to respond to the massive surge in demand for products given limited manufacturing capacity to scale production, and the limited inventory reserves available. The “just-in-time model” led to conservation strategies by GPOs, where AHS was put on “allocation” meaning the GPO was unable to procure additional supply volumes beyond the normal contracted volumes of products. In some instances, suppliers were unable to deliver their normal contracted volumes. The conservation measures relied on allocation formulae that prescribed the supply volumes available to AHS, despite the significant surge in demand of patients and the urgent need for critical supplies in much higher volumes than normal, described in the following:

*“The other piece is that [GPO name] says, well my manufacturers can't supply it to you. [They] can actually say that and get away with that because they are a virtual entity. If I am the distributor for Alberta, I actually can't go to Alberta and say sorry, tough luck, you don’t get masks.”* (Health System Leader)

*“The funny thing that happened was all these distributors came back to us and said you know what, we actually can’t help you, we have no product. We’re putting you on allocation. Every vendor is on allocation.”* (Health System Leader)

Alberta Health services had considerable strengths in a centralized supply chain management for the province that was digitally enabled to create transparency and highly accurate data on supply utilization and demands. However, the global shortages of supplies rapidly escalated, resulting in having no alternative but to find new sources of products in order to meet the surge in demand for critical products to support capacity for care delivery across the province.

### Sourcing and procurement strategies

The CPSM team designed three strategies to find alternative sources of critical supplies in order to manage the massive increase in demand for care: (i) establishing contracts directly with manufacturers; (ii) diversifying supplier sources; and (iii) engaging companies in Alberta as domestic suppliers. Not long after the onset of the first wave of the pandemic, the scope of the mandate of AHS increased to supply PPE to all health organizations and teams (e.g., primary care, long-term care, and private practice settings), as well as a number of community agencies and essential work environments (e.g., borders and airports). This expanded scope was designed to further strengthen the pandemic response across the province; however, it placed additional pressure on the CPSM team to ensure critical supplies were available to all who needed them. Each of the three strategies engaged to source products are described.

(i) **Contracts with manufacturers:** The first strategy implemented to find alternative sources was to mobilize relationships between individuals in Alberta and their network in China, where the majority of manufacturers of PPE were located. The CPSM team contracted with procurement specialists based in China to leverage procurement expertise in China to identify manufacturers to help source direct contract relationships with manufacturers. The CPSM strategy focused on procuring manufacturer capacity, rather than limit procurement efforts to purchasing supplies, described:

*“We did manufacturer direct contracts through an intermediary whereby, we have contracts with two leading manufacturers in China … we actually have to have a source that is going to give us committed production time, and production quantities for about an 18–24-month period so that we can see where the market is going to end up.”* (Supply Chain Leader)

The CPSM team shifted their supply sourcing efforts to focus on manufacturing capacity, rather than compete with every global health system for purchasing supplies.

(ii) **Diversifying suppliers:** The second strategy was the shift from single geographic supplier sourcing to multiple geography suppliers sourcing to offer a balanced supplier network to mitigate risk of supply interruptions. Diversity in supplier sourcing offered redundancy in supply sources so that in the event of further supply shortages or disruptions, Alberta could source product from multiple and varied jurisdictions, described in the following:

*“I think for some items we were very North American centric. For those items, I think we now have a mix of probably Asia and North America. If I look at N95s for example, we are now China and US… we also were able to get 3M masks released through the 3M factory out of Thailand for example. And then, if you look at procedure masks, I think that landscape has shifted from China to very North American centric, right. Gloves, I think, has shifted from a predominantly Malaysia market.”* (Supply Chain Leader)

(iii) **Mobilization of domestic suppliers:** As the second wave unfolded, the CPSM team looked for new sources of domestic manufacturers to offer further sources of critical supply which, overcome distribution logistics from offshore manufacturers. Sourcing of product from manufacturers in China had resulted in some shipments being defective, or inadequate for use due to poor quality (e.g., unusual odour of masks). Domestic suppliers offered the opportunity to work closely with manufacturers to ensure product quality could be assessed locally to ensure product would be acceptable for use. A supply chain leader describes this strategy:

*“The second wave also gave us an opportunity because in the first wave it was, you know, try and find the best supply possible and work with local to see if we can get something done. We actually started working with a local manufacturer for masks for example, and we said ‘well, if you can come up with a mask working with us, we would commit to buying a certain number of masks’... We now have a local manufacturer of masks in Alberta, started off with a volume of four million a month, scaled it all the way up to 10 million (per month) right now.”* (Health System Leader)

Sourcing and procurement approaches rapidly shifted away from the traditional focus on lowest cost (e.g., purchasing through GPO contracts to leverage economies of scale to achieve lowest cost) and moved toward more diverse sourcing strategy focused on contracts with manufacturers, both local and global, to reduce the risk of experiencing critical supply shortages in future.

### Data and digital supply chain infrastructure inform decisions and outcomes

Leaders and decision-makers in Alberta had the advantage of access to real-time supply data to accurately track supply utilization volumes across the province. These data also offered substantial value in tracking the outcomes unfolding across the province. One informant shared the use of supply chain data to track the effectiveness of public health measures, such as transmission of the virus among the health workforce, described in the following:

*“For the PPE updates we were looking at our data from workplace health and safety, like how many of our workers had been infected and where were they getting infected, because they (Decision makers) wanted to know whether the guidelines for wearing PPE should be different in different units. This is about supply chain data informing decisions on outcomes, in this case infection rates relative to use of PPE.”* (Health System Leader)

The digitally-enabled supply chain infrastructure enabled integration of data in near real time to provide insights into pandemic outcomes associated with supply utilization aligned with public health measures to contain the spread of infection. This transparency provided leaders with supply chain data on use of PPE measures, relative to outcomes such as the capacity of hospital beds, ICU capacity, and COVID transmission outcomes. The importance of data-driven decisions is described in the following:

*“I mean we really used the data to actually guide us as to what we were doing. That helped us set the target. I think everything that we decided in terms of the targets and the numbers were based on evidence. It was always based on data and evidence and the Premier was very strong about that.”* (Health System Leader)

Data infrastructure enabled leaders to track COVID-19 outcomes (e.g., rates of transmission of the virus, hospitalization rates, and demands on supply inventory to support care teams and public health measures) relative to warehousing and inventory levels of critical products. The ability to access the data in real time allowed for predictive analytics and modelling to inform procurement teams on sourcing priorities regarding specific supplies needed, product volumes required to support care delivery, and how supply distribution is prioritized. The digital infrastructure in Alberta enabled leaders to make proactive, data-driven decisions on supply procurement and utilization, which resulted in sufficient supply inventory that made it possible for the Alberta government to share supplies with other provinces experiencing critical shortages:

*“The Premier really wanted to make sure that we supported the other provinces and that was when we gave some PPE to Ontario, Quebec, and actually to BC. So that was kind of the story around that, but as I said, there’s no way that we would have been able to do that without the data and the modelling to know how much we need.”* (Supply Chain Leader)

Digitally-enabled supply chain infrastructure was a strategic asset for the province of Alberta that enabled leaders to make data-driven decisions, informed by transparency of supply inventories, track accurate “burn rate” or supply utilization, and track the outcomes of public health measures (e.g., mask mandates) to mitigate the risks of transmission of the virus, particularly among the health workforce. Data emerging from supply chain infrastructure offer the unique opportunity to proactively source and procure products to ensure health organizations have the capacity to effectively and safety respond to rapidly changing events throughout pandemics.

## Conclusion and implications for health leaders

The pandemic response in Alberta was unique to all other provinces, primarily as Alberta is the only centralized, province-wide health system participating in this study, and also due to the most advanced digitally enabled supply chain strategy, which was well-established prior to the onset of the pandemic. The history of emergency preparedness, provincially organized networks of clinician leaders, and proactive strategies to source and procure new models of supply sourcing focused on manufacturing capacity, are key strengths that contributed to Alberta’s capacity to effectively respond to the pandemic. Leadership strategies in this case study highlight important implications for leaders of health systems and are aligned with key dimensions of the LEADS in a Caring Environment framework, which embodies the key skills, behaviours, abilities, and knowledge required to lead in all sectors of the economy and types of organizations.^35^ The findings from this case study reflect leadership capacity aligned with four dimensions of the LEADS framework: Achieving results, Engaging others, Develop coalitions, and System transformation.^35^

### Achieving results

Alberta leaders were able to demonstrate impressive results, by leveraging supply chain data to inform decisions on pandemic management that proactively engaged new sources of PPE, and enabled traceability of outcomes and progress of COVID-19 cases across the province. Data-driven decisions were also critical for accurate modelling and forecasting of supply volume demands that made it possible for Alberta to supply other provinces with PPE. Finally, data were used as critical evidence to evaluate the effectiveness of PPE policy relative to the rates of transmission of the virus to the workforce. Data-driven decisions, informed by a digitally-enabled supply chain infrastructure, was a strategic asset Alberta used to evaluate the effectiveness and impact of pandemic response efforts.

### Engaging others

This was evident in the Alberta case study. Alberta leaders established an emergency management infrastructure well before the first case was diagnosed in Alberta, and engaged a wide range of expertise across the province leveraging the SCNs of leaders that included clinical, academia, and citizen domains of knowledge to inform decision-making. The SCN’s and AHS leaders were able to effectively mobilize knowledge and provincial networks of leaders to inform pandemic management strategies, particularly in the early waves of COVID-19. Leadership decisions were led by government (e.g., cabinet committee), informed by supply chain data from across the province that enabled rapid expansion of supply sourcing strategies to ensure most every organization in the province had access to PPE to mitigate the risk of transmission of the virus.

### Develop coalitions

Alberta findings reveal that this province had existing coalitions (e.g., networks) of experts in the SCNs. The SCNs were mobilized early in Alberta’s pandemic response and served an important role in mobilizing intelligence, expert knowledge, and evidence to support and inform decisions, as well as support and enable implementation of pandemic strategies.

### System transformation

This is a hallmark feature of Alberta’s health system dating back to 2009 when all regions and hospitals were consolidated into a single provincial health system under one administration, AHS, along with implementation of a digitally-enabled supply chain infrastructure, which assumed a critical role in data-driven leadership decisions to support pandemic management. The success of Alberta’s pandemic strategy was further evidenced by its capacity to share inventories of supplies with other provinces in critical need due to inability to procure critical supplies fast enough to meet the surge in demand.

The implications of the Alberta case study for health leaders are:

• Investing in a robust healthcare supply chain digital infrastructure enables leadership decision-making that is grounded in real-time data to achieve proactive responses to events causing disruption in supply, and to inform decisions that are able to proactively manage pandemic supply inventories.
• Data-driven decisions that are able to evaluate and track the effectiveness of decisions, such as public health decisions to prevent transmission of the virus, were centrally important to the success of this province in managing the pandemic early in waves one and two.
• Developing sustained, strategic coalitions, or networks of multisectoral experts, including clinical and healthcare supply chain, enables the mobilization and multidisciplinary understanding of evidence to support sound decision-making.
• Alberta supply chain leadership was able to pivot and adjust their supply chain practices, moving away from traditional supply chain to develop strategies that appear to be more resilient to future disruption. They used domestic suppliers, contracts with manufactures, and diversified suppliers as a means to change and improve their approach.
• Due to its centralized nature, there was a “single source of truth” that created clear, concise communication and provided direction across the system. The clear approach mitigated confusion and allowed for direct lines of accountability, with clear lines of action, to be issued across the province. The lead for pandemic management decisions was the Premier and cabinet, with public health assuming an advisory role. There is evidence that decisions of political leaders in managing the pandemic are influenced by political party values rather than being directly informed by evidence and public health expertise.

Collectively, the strengths of Alberta’s supply chain infrastructure afforded and enabled data-driven decisions based on evidence, which accelerated efforts to strengthen diverse supplier sourcing and mobilize domestic manufacturing. Achieving results, engaging others and mobilizing existing coalitions of expertise, and leveraging a robust digitally enabled supply chain infrastructure were examples of provincial leadership dimensions, which contributed to very resilient supply chain capacity across the province.
